# Effects of 12-week Tai Chi program on physical function, depression, and quality of life among cognitively impaired older adults: a feasibility study

**DOI:** 10.1186/s12877-023-03840-2

**Published:** 2023-03-03

**Authors:** Hyunkyoung Oh, Rhayun Song, Seon Joo Kim

**Affiliations:** 1 University of Wisconsin-Milwaukee, College of Nursing, PO Box 413, Milwaukee, WI 53211 USA; 2 Chungnam National University, College of Nursing, 266 Munwha-ro, Jung-gu, Daejeon, 35015 South Korea; 3 Chungnam National University Hospital, 282 Munwha-ro, Jung-gu, Daejeon, 35015 South Korea

**Keywords:** Tai Chi, Quality of life, Mild cognitive impairment, Dementia, Feasibility

## Abstract

**Background:**

Older adults with cognitive decline need physical activity for maintaining brain health and mitigating cognitive decline. Tai Chi is a safe and gentle aerobic exercise and has been recommended for people with various health conditions to improve their physical functioning, well-being, and quality of life (QoL). This study aimed to determine the feasibility of a 12-week program of Tai Chi for memory (TCM) among older adults with mild cognitive impairment (MCI) or dementia; and to determine the pilot effects of TCM on physical functioning, depression, and health-related QoL.

**Methods:**

A quasi-experimental design was used with two groups: MCI and dementia. The feasibility of the 12-week TCM program was assessed after it finished in terms of its acceptability, demand, implementation, practicality, adaptation, integration, expansion, and limited-efficacy testing. Other health-related outcomes, physical functioning, depression, and health-related QoL were measured before and after the TCM program. Outcome measures consist of a digital hand dynamometer for grip strength, the standard sit-and-reach test, the one-leg-standing balance test, timed up and go (TUG) test, the Korean version of the Geriatric Depression Scale, and the 12-item Short Form survey (SF-12). Paired and independent *t*-tests were used to compare the effects of TCM within and between groups.

**Results:**

The TCM program was completed by 41 participants with MCI (*n* = 21) or dementia (*n* = 20), and its accepted feasibility was assessed. After TCM, the MCI group exhibited significant enhancements in right-hand grip strength (*t* = − 2.13, *p* = .04) and physical-health-related QoL (*t* = − 2.27, *p* = .03). TUG scores improved in both groups (MCI, *t* = 3.96 *p* = .001; dementia, *t* = 2.54 *p* = .02). The adopted form of the TCM program was effectively and safely applied to those with various levels of cognitive impairment. The program was well accepted by the participants with a mean attendance rate of 87%. No adverse events were reported during the program.

**Conclusion:**

TCM has the potential to improve physical functioning and QoL. Since there was no comparison group to control for confounding factors and low statistical power in the present study, further studies are warranted with a stronger design that includes longer follow-up periods. This protocol was retrospectively registered on Dec 1, 2022 (NCT05629650) at ClinicalTrials.gov.

## Background

Globally, 6.6% of people aged 60 years and older have mental and neurological disorders including Alzheimer’s disease (AD) and dementia [1]. More than 55 million people live with dementia worldwide, and there are nearly 10 million new cases every year [2]. Dementia has significant social and economic impacts on patients and their families [3]. The estimated total global cost of dementia was $1.3 trillion [2]. The health outcomes and quality of life (QoL) of patients with cognitive disorders and their caregivers are also notably worse than their age-matched peers without neurocognitive disorders [3].

Pharmacological and nonpharmacological treatments are used to maintain and reduce cognitive decline. Specifically, nonpharmacological interventions are preferred for patients with mild cognitive impairment (MCI), which is a stage of predementia [4]. Among nonpharmacological interventions, physical activity is a promising method for not only improving cognitive functioning and QoL among people with MCI [5], but also for reducing depressive symptoms and enhancing cognitive functioning and QoL among people with dementia [6–8]. Major health organizations such as WHO, American Heart Association, and Alzheimer’s Association, also recommend physical activity for maintaining brain health and mitigating cognitive decline [3]. Moreover, these health organizations recommend treating cardiovascular disease and risk factors [3]. There is a considerable amount of evidence that physical activity is beneficial for the cardiovascular system in young, old, healthy, and diseased populations due to it decreasing cardiovascular risk factors and improving cardiorespiratory fitness levels [9, 10]. Current evidence also supports that the presence of cardiovascular disease and its risk factors elevate the incidence rates of both vascular cognitive impairment and AD [11, 12]. Declined cardiovascular function plays a crucial role in the acceleration of cognitive deterioration by worsening cerebral perfusion and blood pressure control, and promoting disturbances in amyloid clearance [11, 13]. Increased physical activity is therefore required for people with cognitive disorders to improve cardiovascular function and mitigate cognitive decline.

Tai Chi is a safe and gentle aerobic exercise that addresses the mind, body, and spirit. This traditional mind-body exercise is relatively nonstrenuous, low impact, and includes relaxation, meditation, and deep and regulated breathing techniques, and slow graceful movements [14]. As a type of physical activity, there is evidence that Tai Chi improves aspects of physical functioning such as balance, flexibility, cardiorespiratory fitness, and agility [15–17], and other functions such as well-being and QoL [18]. Due to these clinical benefits, Tai Chi has been recommended for people with various health problems such as musculoskeletal conditions, cardiovascular diseases, cancer, and mental disorders to improve their physical functioning, well-being, and QoL [19–22]. There is also evidence that Tai Chi has positive clinical effects on cognitive functioning among older adults with MCI [23] and dementia [24]. However, it is unclear whether Tai Chi also has positive effects on physical functioning and QoL among this population.

In this study, we used the Tai Chi for memory (TCM) program. TCM was developed for people with cognitive impairment [25] while considering the characteristics of older adults with cognitive impairment such as limited ability to recall or follow movements and increased fall risk. TCM consists of 14 movements from the Yang and Sun styles of Tai Chi, which provide blocked sets of main features with repeating movements to make it easier for participants with memory difficulties [26]. However, the feasibility of TCM was not examined yet. This study aimed to apply TCM to community-residing older adults with MCI or dementia for 12 weeks and to examine the feasibility and pilot effects of TCM on physical functioning, depression, and QoL. The specific objectives of the study were as follows:

1. To identify the feasibility of TCM for older adults with MCI or dementia for 12 weeks, including in terms of its acceptability, demand, implementation, practicality, adaptation, integration, expansion, and study limitation.
2. To compare the effects of TCM on physical functioning (grip strength, balance, flexibility, and mobility), depression, and health-related QoL among older adults with MCI and dementia.

## Methods

### Design

This quasi-experimental pretest-posttest study without a control group examined the feasibility of 12-week TCM for two groups of older adults with cognitive impairment, and compared the effects of TCM on physical functioning, depression, and the health-related QoL within and between the MCI and dementia groups.

### Participants

**Fig. 1:**
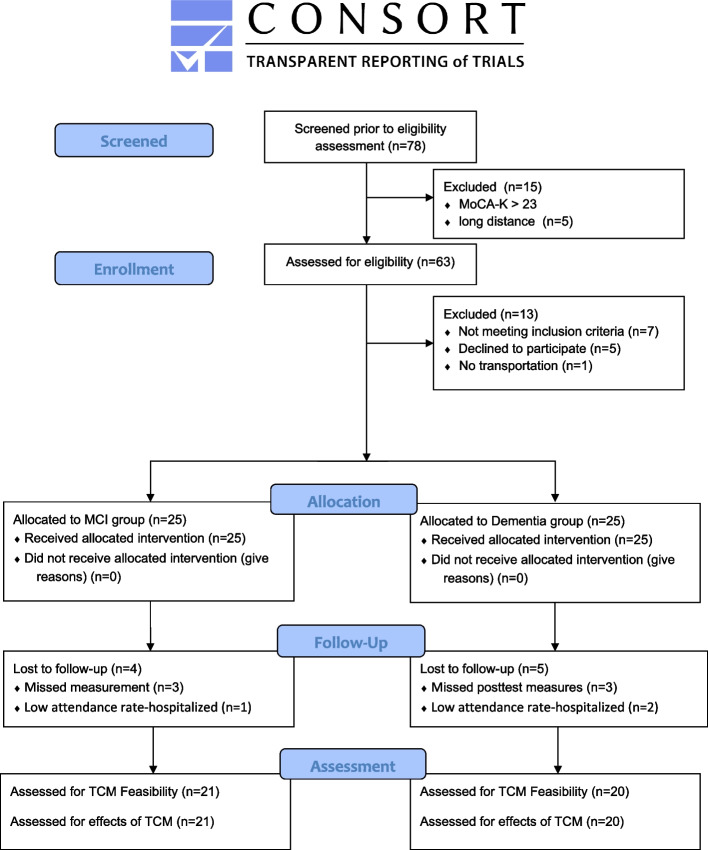
Flow Diagram. Based on CONSORT 2010 statement: extension to randomized pilot and feasibility trials [28]

Older adults were recruited from three public dementia prevention centers in rural areas of South Korea. These centers were selected based on similarities in population sizes and screening procedures used in dementia prevention programs. The inclusion criteria were (1) community-residing adults aged 75 years or older, (2) registered at the public dementia prevention centers for dementia screening by health professionals with the diagnosis of either MCI or dementia, (3) Korean version of the Montreal Cognitive Assessment (MoCA-K) score of 22 or lower, (4) had not participated in any formal regular exercise program during the previous 6 months, and (5) agreed to participate in the Tai Chi program twice a week for 12 weeks. Those with musculoskeletal disorders, other neurological/psychiatric diseases other than dementia, or chronic conditions that would prevent them from participating in regular exercise were excluded. The recruitment period was from July 2018 to March 2019. According to Julious, it is recommended that a minimum of 12 subjects per group be considered for pilot studies in terms of feasibility and precision [27]. Considering the rule and the possible dropout rates of the pilot intervention study, we recruited participants with MCI (*n* = 25) or dementia (*n* = 25) to form the intervention groups (Fig. 1).

### Tai Chi intervention

TCM is a standardized type of Tai Chi for health developed by Dr. Lam specifically for those who experience or want to prevent cognitive problems [29]. As a group exercise, TCM was provided at 60 minutes/session, twice a week for 12 weeks at the community center by a certified Tai Chi instructor experienced in teaching older adults with cognitive impairments over 10 years. Although there is no required equipment for demonstrating TCM, an individual chair was provided during the session to sit in while learning the movements because most participants used a cane or assisting device to walk around. All participants with a chair were allowed to sit at any time if they felt tired during the standing Tai Chi movements. The adapted form of TCM, consisting of 12 Sun and Yang Tai Chi style movements, can be provided while sitting and standing with five blocked movement sets so that cognitively impaired individuals are able to follow them. Tai Chi walking was practiced while sitting to practice weight transfer, and then while standing to practice moving forward and backward. The intensity of Tai Chi movements progressively increased from mostly sitting to less sitting and more standing. The 60-min session consisted of 5 min of greetings among group members, a 5-min warm-up, 40-min main exercise, 5-min cool-down, and 5-min greetings and homework exercises. The same TCM program applied to both groups. One research team member made phone calls to participants to remind their sessions and the member tracked each session attendance of participants. The protocol was registered at ClinicalTrials.gov (NCT05629650).

### Feasibility monitoring

An experienced Tai Chi instructor led the program, while the research staff monitored for any adverse effects or fall risks during the sessions. The feasibility of the program was assessed in terms of acceptability, demand, implementation, practicality, adaptation, integration, expansion, and efficacy study limitation [29].

### Measurement

Outcome measurements were performed at the baseline and the posttest after the 12-week TCM program by a trained research team. A structured questionnaire comprised a face-to-face interview to measure depression and QoL as well as sociodemographic information. Physical functioning was assessed by another research team who did not know the group of participants.

#### Physical functioning

##### Grip strength

Grip strength was assessed in both hands using a digital hand dynamometer (0–90 kg; Labisen KS-301). The higher score of two repeated measurements was recorded for each hand.

##### Flexibility

The standard sit-and-reach test (SSRT) [30] was used to assess the flexibility of the lower back and hamstrings. Participants sat on the floor with their legs out straight toward the makeshift box with a ruler attached and knees flat against the floor, while leaning forward slowly as far as possible and holding this position for at least 2 seconds. The distance between the stretched fingertips and the box was measured. Since negative scores indicated not reaching the box, and positive scores for going beyond the edge of the box, higher scores (lengths in centimeters) represented better flexibility.

##### Balance

Balance was assessed using a one-leg-standing balance test (OLST) [31]. The eyes-open OLST determined for how long the participants could cross their arms while standing on 1 foot of their choice. Higher scores (measured in seconds) indicated better balance.

##### Mobility

Mobility was assessed by the Timed Up and Go test (TUG), which measured the time that it took for the participants to stand up from a chair, walk to a line on the floor 3 m away, turn around, and walk back to the chair and sit down at a normal pace [32]. Lower scores (in seconds) indicated better mobility.

#### Depression

Depression was assessed by the short form of the Korean version of the Geriatric Depression Scale (SGDS-K) [33]. SGDS-K comprises 15 items; and has been validated in the Korean elderly population (cutoff point of 16). Its Cronbach’s alpha was 0.88 and test-retest reliability was 0.75. Higher scores indicated more depressive symptoms.

#### Quality of life

QoL was measured using the SF-12 [34], which consists of eight dimensions: general health, physical functioning, role physical, body pain, vitality, social functioning, role emotional, and mental health. We obtained permission from QualityMetric Inc. and administered the questionnaire through a face-to-face interview. We calculated two summary scores for the physical and mental components of health-related QoL using the weighted means for the eight domains. Higher scores indicated better health-related QoL.

### Data collection

Data collection took place in three centers simultaneously. Individual interviews were conducted to assess sociodemographic information, depression, and health-related QoL. The trained research staff measured grip strength, balance, flexibility, and mobility according to the standard protocol.

### Analysis

Descriptive statistics were used to analyze the sociodemographic characteristics and outcome variables of both groups using SPSS 25.0. The effects of the Tai Chi program on physical functioning, depression, and QoL were compared within groups using paired *t*-tests, and an independent t-test was used to compare differences in mean scores between the two groups. All outcome variables were analyzed at the 0.05 alpha level. The statistical assumption of normality for continuous variables was assessed by skewness or kurtosis above an absolute value of 2.0, and a bootstrap with 1000 samples was used for the analysis considering the small sample size.

### Ethical considerations

This study was approved by the Institutional Review Board of the Chungnam National University Hospital (CNUH 2018–07-070) where the primary researcher was affiliated. The dementia prevention centers were contacted to obtain permission. The research staff obtained the written consent form from each participant after explaining the purpose of the study, its protocol, the confidentiality of the data, safety information regarding benefits and risks, and the possibility of withdrawing at any time without any consequences.

## Results

### Study feasibility

#### Acceptability

TCM was well accepted by the participants. At the end of the program, the participants expressed that they enjoyed coming to the class and felt that they belonged to the group while practicing together.

#### Demand

The participants completed TCM with an average attendance rate of 87% (ranging from 40 to 100), with absentees mostly citing family circumstances. There were no adverse events reported by the participants during the program.

#### Implementation

A standardized TCM protocol was implemented. The protocol included greetings, a warm-up to enhance Qi and balance, main movements from sitting to standing in a block system, followed by cool-down with gentle stretching, and Dan Tian breathing. The order of the protocol was implemented consistently during each session, while the duration of practicing main movements from sitting to standing varied to assess the progress of the participants in each group.

#### Practicality

Progress in learning the main Tai Chi movements in blocks was slower for those with dementia than for those with MCI, but all participants in both groups were able to follow the protocol until the end of the study period. The participants were allowed to be excused from the class when they felt tired or distracted, and individual chairs were provided.

#### Adaptation

The physical and cognitive functioning of the participants was considered when implementing the TCM program. Strategies that adapted Tai Chi to this population were implemented in the TCM program. First, the movements of TCM were selected based on the features of being simple and safe to perform as well as effective for implementing Tai Chi principles into the movements. Second, each session was provided based on the protocol including three or more repetitions of blocked TCM movement sets accompanied by meditation music, so that the structure of sessions was consistent throughout the study period, making the participants feel comfortable in following it. Third, the Dan Tian breathing exercise was a good way to allow the participants to rest in chairs while practicing movements that matched breathing patterns. Fourth, one of the most important features of TCM in improving the balance and mobility of this population is the Tai Chi walking exercise. This could be performed while sitting to learn the principles without weight transfer, then moving to a standing position to practice walking with weight transfer from empty to full steps.

#### Integration

TCM is a mind-body exercise that combines the features of aerobic exercise (warm-up, main exercise, and cool-down) with Tai Chi principles (Dan Tian breathing, Qigong, and meditation). The intensity and implementation of the TCM program were also adapted to adhere to the limited physical and cognitive functioning of older adults.

#### Expansion

We applied the TCM program to a population with various cognitive and physical abilities. This program was effectively and safely applied to this population with no adverse events reported, which indicated its potential of being expanded to others with similar functional limitations.

#### Limited-efficacy testing

A clinical efficacy of TCM was evaluated. The findings indicated potential effects on physical functioning and QoL among this population, but there was no comparison group to control for confounding factors, and consequently, the statistical power was relatively low. Further studies are warranted with a stronger design that includes longer follow-up periods.

### General characteristics of the participants

The participants with a diagnosis of either MCI (*n* = 21) or dementia (*n* = 20) had a mean age of 79 years and were mostly female (90–95%), had no education experience (52–55%), widowed (61–85%), and had a low economic status (90–95%). Most of them were able to perform independent daily activities, but 9–20% of them required some assistance. Most participants had multiple chronic conditions, including arthritis, hypertension, or diabetes. The mean scores for cognitive functioning assessed using the MoCA-K were 17.05 and 9.20 in the MCI and dementia groups, respectively (Table 1).

**Table 1:** Demographic characteristics of the participants with mild cognitive impairment and dementia

Variables	Categories	MCI Group (*n* = 21)	Dementia Group (*n* = 20)	χ^2^ or *t*	*p*
Age		79.76 (4.04)	79.30 (7.27)	0.25	.80
Attendance rates		86.47 (7.92)	87.00 (7.50)	−0.21	.82
Sex	Male	1 (4.8)	2 (10.0)	0.41	.60
	Female	20 (95.2)	18 (90.0)		
Education (years)	None	11 (52.4)	11 (55.0)	2.92	.40
	1 ~ 6	7 (33.3)	4 (20.0)		
	6 ~ 12	3 (13.3)	5 (25.0)		
Marital status	Married	8 (38.1)	3 (15.0)	2.78	.16
	Widowed	13 (61.9)	17 (85.0)		
Income	Low	20 (95.2)	18 (90.0)	1.08	.58
	Middle or high	1 (4.8)	2 (10.0)		
ADL	Independent	19 (90.5)	16 (80.0)	0.90	.34
	Assistance required	2 (9.5)	4 (20.0)		
Comorbidity	Hypertension	10 (47.6)	10 (50.0)	0.02	.89
	Cardiovascular disease	2 (9.5)	1 (5.0)	0.39	.57
	Diabetes	5 (23.8)	4 (20.0)	0.09	.76
	Stroke	3 (14.3)	3 (15.0)	0.00	.95
	Arthritis	16 (76.2)	12 (60.0)	1.24	.26

Data are n (%) or mean (SD) values

*ADL* activities of daily living

### Effects on physical functioning between MCI and dementia

Physical functioning was assessed based on grip strength, flexibility, balance, and mobility. The MCI group had significantly increased right grip strength (mean = 19.31 to 20.38, *t* = − 2.13, *p* = .04) and enhanced mobility (mean = 9.64 to 7.50, *t* = 3.96, *p* = .001) after 12 weeks of TCM. The dementia group had significantly increased mobility (mean = 13.05 to 10.70, *t* = 2.54, *p* = .02) after 12 weeks of TCM (Table 2). There were no differences between the two groups before and after the intervention period.

**Table 2:** Changes in study outcomes over 12 weeks between the MCI and dementia groups (*N* = 41)

Variables	MCI (*n* = 21)				Dementia (*n* = 20)				differences in mean			
	pretest	posttest	paired t	*p*	pretest	posttest	paired t	*p*	MCI	Dementia	*t*	*p*
	Mean (SD)	Mean (SD)			Mean (SD)	Mean (SD)			Mean (SD)	Mean (SD)		
MOCA	17.05 (2.80)	17.14 (4.57)	−0.09	.925	9.20 (3.07)	9.45 (3.85)	−0.38	.702	−0.09 (4.54)	0.01 (3.07)	−0.07	.938
grip_Lt	18.95 (5.77)	19.47 (5.10)	−0.91	.372	17.45 (5.40)	17.00 (6.16)	0.35	.729	0.01 (2.62)	0.45 (5.72)	−0.32	.746
grip_Rt	19.31 (5.79)	20.38 (5.11)	**−2.13**	.045	18.45 (4.68)	19.60 (6.57)	−1.36	.188	− 1.07 (2.27)	− 1.12 (3.77)	0.05	.956
TUG	9.64 (3.55)	7.67 (1.77)	**3.96**	.001	13.05 (8.03)	10.70 (6.66)	**2.54**	.020	1.96 (2.27)	2.34 (4.13)	−0.36	.714
SSRT	11.40 (9.91)	9.78 (8.49)	0.99	.333	6.08 (10.61)	7.11 (7.19)	−0.60	.551	1.61 (7.48)	−1.02 (7.57)	1.12	.267
OLST	2.69 (1.76)	2.71 (1.70)	−0.04	.965	2.00 (2.05)	2.11 (1.74)	−0.30	.764	−0.02 (2.34)	−0.10 (1.60)	0.13	.892
Depression	4.23 (4.35)	4.42 (4.80)	−0.24	.809	5.85 (5.01)	5.00 (4.29)	0.99	.332	−0.19 (3.57)	0.85 (3.81)	−0.90	.373
PCS	38.43 (12.89)	42.55 (10.60)	−**2.27**	.034	43.00 (11.21)	42.42 (9.36)	0.29	.773	−5.07 (10.20)	0.58 (8.88)	−1.88	.066
MCS	47.23 (10.85)	49.02 (12.27)	−0.98	.338	43.47 (15.93)	46.78 (12.29)	−1.92	.069	−1.78 (8.31)	−3.31 (7.68)	0.61	.544

Data are mean (SD) values

*MoCA-K* Korean version of the Montreal Cognitive Assessment, *TUG* Timed Up-and-Go test, *SSRT* standard sit-and-reach test, *OLST* one-leg-standing balance test, *PCS* physical-components score of the health-related QoL, *MCS* mental-components score of the health-related QoL

### Effects on depression between MCI and dementia

The baseline depression score did differ significantly between the two groups (Table 2). The 12-week TCM did not have a positive effect on depression in either group. The dementia group had decreased depression after the intervention period, but the changes were not significant.

### Effects on health-related QoL between MCI and dementia

QoL was measured using the SF-12, and the two summary scores of the physical components of health-related QoL (PCS) and mental components of health-related QoL (MCS) were calculated using the weighted means for the eight domains. The baseline PCS and MCS did not differ significantly between the groups. The PCS in the MCI group increased significantly after the intervention (mean = 38.43 to 42.55, *t* = − 2.27, *p* = .03). The MCS in both groups showed a tendency to improve after the intervention, but the improvement was not significant (Table 2).

## Discussion

This study aimed to evaluate the feasibility and pilot effects of the TCM program in community-dwelling older adults with cognitive impairment. TCM is a standardized form developed for those with cognitive impairment with specific features so that the participants would feel easy to follow the program. We provided the TCM to two intervention groups with MCI or dementia for 12 weeks to confirm if the adopted form of TCM would be applicable and effective even for older adults with different levels of cognitive impairment.

### Feasibility of TCM

The feasibility of this pilot study was evaluated using eight categories. TCM was accepted by the participants, and they expressed their satisfaction with TCM. Slow movements, applying a block system to learn movements, and providing an individual chair for TCM were implacable, practicable, and adaptable for the participants. Thus, the mean attendance rate was 87% for 12 weeks, and there were no adverse events. A pilot clinical efficacy of TCM was also evaluated. The findings indicated potential effects on physical functioning and QoL among older adults with cognitive impairment however, we found a few considerable issues for future trials.

The first issue is the duration of the program. The 12-week duration provided sufficient time for those in both groups to learn 12 movements and Qigong breathing, but those in the dementia group learned more slowly. A systematic review found that previous studies that applied Tai Chi to people with cognitive impairments mostly used short-form or simplified forms of Tai Chi with durations of 12 weeks to 12 months [23]. Another systematic review of Tai Chi involving patients with dementia also mostly applied the short form of the Sun or Yang Tai Chi styles with durations of 8 weeks to 12 months [24]. Based on the reviews, the duration of the Tai Chi program should be adjusted based on the ability of the participants to learn and perform the movements and principles of Tai Chi for sufficiently long to yield beneficial effects.

The second issue relates to safety in this pilot study, no serious adverse events were reported in either group. However, adverse events were previously observed in studies that applied Tai Chi to individuals with cognitive impairment, in which three of them found unrelated bone fractures [23]. Additionally, there has been concern about the safety of Tai Chi due to poor and inconsistent reporting of adverse effects [35]. Tai Chi is considered relatively safe, but safety-monitoring protocols should be clearly described especially for individuals with limitations to physical and cognitive functioning.

### Pilot effects of TCM

This study found that TCM significantly improved right-hand grip strength, mobility, and physical-health-related QoL in older adults with MCI. There were no significant differences in balance, flexibility, cognitive functioning, depression, and mental health-related QoL, before, and after TCM. Among the older adults with dementia, TCM only had a positive effect on mobility and did not affect other physical and cognitive functioning, depression, or health-related QoL.

#### Tai Chi and physical functioning

Muscle strength in both hands mostly increased after the 12-week TCM program in both groups. However, there were no significant differences within the groups except for the right hand in the MCI group. This result was consistent with Kim et al. finding no effect on grip strength among adults with Parkinson’s disease and MCI (*p* = .337) [36]. Another study similar found no significant difference in grip strength (*p* = .910) among elderly females with dementia after the 12-week Tai Chi program (60 minutes, three times/week) [37]. It has been reported that Tai Chi has the potential to increase joint flexibility and muscle strength due to its characteristics of emphasizing sequential movements performed with alternate flexion and extension of joints, changes in limb movement directions, dynamic weight shift, and single limb support [15, 38]. However, the age of the present participants (mean > 79 years, Table 1) and duration of TCM (60 minutes, twice/week, 12 weeks) might explain the lack of significant difference in muscle strength found in this study. Indeed, So et al. found that muscle strength was improved among older adults after 24 weeks of Tai Chi exercise [39].

Mobility measured using TUG was significantly improved in both the MCI and dementia groups (*p* = .001 and *p* = .020 respectively). This result was consistent with those of a previous study by Choi et al. on fall prevention in older adults [40]. Although Choi et al. targeted older adults without cognitive impairment, the 12-week Tai Chi program had a positive effect on mobility (*p* = .01). A meta-analysis by Liu et al. [41] also found that groups of older adults with Parkinson’s disease who participated in Tai Chi demonstrated a significantly reduced completion time in the TUG (*p* < .05). On the other hand, some studies involving the elderly with dementia have found no differences in mobility after 20-week Tai Chi consisting of weekly classes and home practice [42, 43]. Tai Chi affects balance control and improves posture sway, and it therefore has the benefit of reducing the time taken in the TUG. However, when considering some key factors associated with adherence to physical exercises such as supervision, communication, feedback, and good group dynamics [44, 45], the improved mobility may be related to increased practice hours.

Increases and decreases in flexibility measured using SSRT and in balance measured using the OLS varied, and the differences were not significant in both groups. These results are similar to those of other studies that applied Tai Chi. Kim et al. found that there were no differences in flexibility (*p* = .198) and balance (*p* = .139) after the 12-week Tai Chi program among adults with Parkinson’s disease and MCI [36]. Yu et al. also found no difference in balance (*F* = 0.30) after an 8-week Tai Chi program in elderly individuals with dementia [46]. In contrast, some other studies found that Tai Chi has a significant effect on balance (*p* < .05) after older adults with MCI [47] performed Tai Chi for a year, and a positive impact on flexibility (*p* < .001) was found after a 12-week Tai Chi program among elderly females with dementia [37]. One of the possible reasons for these inconsistent results is the age (mean > 79 years) and physical condition of arthritis (76% with MCI; 60% with dementia) of the participants in the present study. Joint pain or lack of muscle mass can negatively affect knee flexion and weight shifts that would improve flexibility and balance. Another reason could be related to one of the measurement tools. Balance was measured in seconds, but requires a small variance to measure the difference accurately; using a more-sensitive measurement tool is recommended for older adults.

#### Tai Chi and depression

There were no significant changes in depression scores in both groups. The findings were not supported by previous studies that applied the 12-week Tai Chi to older adults with MCI or mild dementia [48, 49]. Tai Chi involves low-intensity exercises and has effects similar to those of meditation, suggesting that Tai Chi can reduce tension, depression, and anxiety [50]. Indeed, three systematic review articles found that most Tai Chi exercises have a significant effect on depression among older adults [18, 20, 22]. However, those reviews did not include populations with MCI or dementia.

While Tai Chi utilizes the principles of meditation and mindfulness through the Qigong breathing exercise, these benefits for depression were not clear in the present population. The non-significant changes in depression in the present study may be due to the relatively short duration of intervention that may not be sufficient to learn for individuals with MCI and dementia. In addition, the participants were small and there was no control group in the current study. Further studies with larger samples are therefore recommended to confirm the effect of Tai Chi on depression in the MCI and dementia populations.

#### Tai Chi and quality of life

QoL was measured using the SF-12 and calculated as PCS and MCS. QoL in the MCI group improved after the 12-week TCM, and PCS improved significantly (*p = .*034). This was similar to Siu and Lee’s finding that older adults with MCI had improved PCS (*p* = .036) and MCS (*p* = .014) after a 16-week Tai Chi program [51]. However, Xu et al. found that the 12-week Tai Chi program did not impact QoL (*p* = .465) among older adults with MCI [49]. For the dementia group, the PCS decreased but the MCS increased. In contrast, Nyman et al. found that people with dementia that participated in Tai Chi had significantly higher QoL (mean difference = 0.051, effect size = 0.51) [42]. The literature indicates that QoL is improved after Tai Chi among older adults and people with cardiovascular diseases [18, 20, 22]. However, a systematic review found that a group with cancer that performed Tai Chi did not have improved QoL [52]. Regular exercise and emotional support by other group participants can generally enhance the self-efficacy of older adults, which improves their emotional well-being and mental QoL [53]. However, the effect of Tai Chi on QoL may not be consistent according to the different studied populations; further studies with MCI and dementia populations are recommended.

### Implications and recommendations for the future direction

This study tested the feasibility and pilot clinical efficacy of TCM among older adults with cognitive impairments. TCM was examined to be feasible and have the potential to improve physical functioning and QoL. Health care providers in the rural area will offer TCM as a community-based exercise program. However, there are some recommendations for implication of TCM and future studies. First, the longer intervention period will be considered for adults with dementia. This pilot study provided a 60-min session per week for 12 weeks. As we mentioned above, the dementia group needed more time to learn the whole TCM movements compared to the MCI group. Also, a 24-week Tai Chi program reported a significant improvement among older adults. Thus, we recommend a longer intervention period or increased dosage. Second, health care providers and researchers should consider the unexpected synergies among multiple chronic conditions including cognitive impairments, arthritis, geriatric syndromes, sarcopenia, and so on. They can be confounders related to physical outcomes. Third, the intervention team should develop a partnership with caregivers of the participants. As a group and in-person exercise program, the participants needed support from their caregivers to travel to the community center. Thus, good partnership with caregivers is recommended to improve adherence to the intervention. The final recommendation is related to the sensitivity of measurement instruments. When measuring physical outcomes, more-sensitive measurement tools will be recommended to capture a small difference for this population.

### Limitations

The TCM was applied to those with MCI or dementia, but our participants were limited to those who were able to perform activities of daily life (ADL) with or without assistance. Since most participants (90% for MCI group and 80% for dementia group) were independent in performing ADL, the applicability of the TCM is not confirmed with those who require assistance in most ADL, hence leading to the limited generalizability of the program feasibility. In terms of the design, there was no control group in this study to verify the pilot efficacy of TCM, and each group has a relatively small sample size. This could have made it difficult to detect the effects of the 12-week TCM program on physical functioning, depression, and QoL. Future studies with larger samples and stronger designs with control groups are needed to confirm the effects of TCM.

## Conclusion

The feasibility of TCM was considered feasible for older adults with MCI or dementia. TCM also had significant and positive effects on mobility and health-related QoL. We therefore believe that TCM has the potential to improve physical functioning and QoL. Health-care providers working in MCI and dementia populations can apply TCM to their patients to enhance their physical functioning and QoL. To verify the effects of TCM on cognitive functioning, depression, and well-being, health-care providers should consider the age of the participants, sample sizes, TCM frequency and duration, and measurement tools.

## Data Availability

The datasets used and analyzed during the current study are available from the corresponding author upon reasonable request.

## Abbreviations

TCM: Tai Chi for memory
QoL: Quality of life
MCI: Mild Cognitive Impairment
AD: Alzheimer’s disease
