# Correction to: Effects of 12-week Tai Chi program on physical function, depression, and quality of life among cognitively impaired older adults: a feasibility study

**DOI:** 10.1186/s12877-023-03999-8

**Published:** 2023-05-17

**Authors:** Hyunkyoung Oh, Rhayun Song, Seon Joo Kim

**Affiliations:** 1 College of Nursing, University of Wisconsin-Milwaukee, PO Box 413, Milwaukee, WI 53211 USA; 2 College of Nursing, Chungnam National University, 266 Munwha-ro, Jung-gu, Daejeon, 35015 South Korea; 3 Chungnam National University Hospital, 282 Munwha-ro, Jung-gu, Daejeon, 35015 South Korea

**Correction to: BMC Geriatrics (2023) 23:118**

10.1186/s12877-023-03840-2

After publication of this article [1], the authors reported that Hyunkyoung Oh was incorrectly denoted as the corresponding author, but it should have been Rhayun Song.

The original article [1] has been corrected.
